# Individual Scores for Associative Learning in a Differential Appetitive Olfactory Paradigm Using Binary Logistic Regression Analysis

**DOI:** 10.3389/fnbeh.2021.741439

**Published:** 2021-09-28

**Authors:** Kim J. Borstel, Paul A. Stevenson

**Affiliations:** Department of Physiology of Animals and Behaviour, Institute of Biology, Faculty of Life Sciences, Leipzig University, Leipzig, Germany

**Keywords:** animal personality, binary logistic regression, cognition, conditioning, invertebrates, memory, video-tracking

## Abstract

Numerous invertebrates have contributed to our understanding of the biology of learning and memory. In most cases, learning performance is documented for groups of individuals, and nearly always based on a single, typically binary, behavioural metric for a conditioned response. This is unfortunate for several reasons. Foremost, it has become increasingly apparent that invertebrates exhibit inter-individual differences in many aspects of their behaviour, and also that the conditioned response probability for an animal group does not adequately represent the behaviour of individuals in classical conditioning. Furthermore, a binary response character cannot yield a graded score for each individual. We also hypothesise that due to the complexity of a conditioned response, a single metric need not reveal an individual's full learning potential. In this paper, we report individual learning scores for freely moving adult male crickets (*Gryllus bimaculatus*) based on a multi-factorial analysis of a conditioned response. First, in an absolute conditioning paradigm, we video-tracked the odour responses of animals that, in previous training, received either odour plus reward (sugar water), reward alone, or odour alone to identify behavioural predictors of a conditioned response. Measures of these predictors were then analysed using binary regression analysis to construct a variety of mathematical models that give a probability for each individual that it exhibited a conditioned response (*P*_resp_). Using standard procedures to compare model accuracy, we identified the strongest model which could reliably discriminate between the different odour responses. Finally, in a differential appetitive olfactory paradigm, we employed the model after training to calculate the *P*_resp_ of animals to a conditioned, and to an unconditioned odour, and from the difference a learning index for each animal. Comparing the results from our multi-factor model with a single metric analysis (head bobbing in response to a conditioned odour), revealed advantageous aspects of the model. A broad distribution of model-learning scores, with modes at low and high values, support the notion of a high degree of variation in learning capacity, which we discuss.

## Introduction

A wealth of studies have established the value of invertebrates, and particularly insects for understanding the fundamental principles underlying learning and memory (reviews—Drosophila larvae: Gerber and Stocker, 2007; various: Leadbeater and Chittka, 2007; honey bees: Menzel, 2012; cockroaches and crickets: Mizunami et al., 2013; paper wasps: Tibbetts and Sheehan, 2013; Giurfa, 2015; Drosophila: Boto et al., 2020). In the vast majority of studies, learning is typically reported as the number of individuals in a group that show a conditioned response. For example, as the proportion of animals that either move actively towards a conditioned stimulus (Drosophila larvae: Neuser et al., 2005; Saumweber et al., 2011), or exhibit a readily identified conditioned response, such as the proboscis extension reflex (PER) in honey bees (Bitterman et al., 1983). However, in honey bees, the gold standard in insect learning, it has become evident that the conditioned response probability of an animal group does not adequately represent the behaviour of individuals in classical conditioning (Pamir et al., 2011). Thus, while a group score is perfectly suitable for most applications, the spectrum of individual performances, and with it an opening to understand their causes, are overlooked. This is regrettable considering that one of the most influential findings in the field of animal behaviour of recent years is that members of the same species show consistent inter-individual differences in specific behaviours, with suites of traits that correlate positively across time and different contexts to form a behavioural syndrome (review: Sih and Bell, 2008; Wilson et al., 2019). The intriguing possibility that variation in cognition is coupled to other traits in a “cognitive syndrome” has been discussed at length, but there is still little supportive evidence in non-human animals (Sih and Del Giudice, 2012).

It is nonetheless well-documented, that individual learning performance can vary considerably in vertebrates (e.g., Dukas, 2004; Groothuis and Carere, 2005) and there are several reports of this in invertebrates, including insects (Dukas, 2008). The existence of individual variation has been inferred from selective breeding for poor and good learners (blow flies: McGuire and Hirsch, 1977), or by showing consistency in performance of groups with differing learning capacity over successive trials (honey bees: Pamir et al., 2011, 2014; cockroaches: Arican et al., 2020). Measures of actual individual performances are difficult, since they are typically based on a binary choice, i.e., whether or not an animal exhibits a specific behaviour as a conditioned response, such as extending its mouthparts in appetitive learning trials (honey bees: Bitterman et al., 1983; ants: Guerrieri and D'Ettorre, 2010; cockroaches: Arican et al., 2020), or choosing the correct path in a Y-maze (Giurfa et al., 1999; Dupuy et al., 2006). Estimates of learning capacity in individual focal animals have nonetheless been achieved. For example, by evaluating the relative portion (Drosophila larvae: Neuser et al., 2005; Saumweber et al., 2011), or percentage (bumble bee: Muller and Chittka, 2012) of correct choices in multiple trials, or the time spent probing a conditioned stimulus (Matsumoto and Mizunami, 2002), or time taken for choice behaviour (Scheiner et al., 2020). In all such cases, proficiency is assessed from a single behavioural metric, which, given the complexity of conditioned responses (Onodera et al., 2019), need not embrace an individual's full response potential.

In the present paper, therefore, we report individual learning scores for freely-moving male crickets, *Gryllus bimaculatus*. These insects have become a focus for studies of aggression (review: Stevenson and Rillich, 2019) and how experiencing aggression during development forges life-long inter-individual differences in adult behaviour (Rose et al., 2017; Balsam and Stevenson, 2020, 2021). Earlier studies have also demonstrated the aptitude of crickets for studying learning and memory (review: Mizunami and Matsumoto, 2017). Basically, two different methods have been applied: a mixed appetitive—aversive, differential conditioning procedure to evaluate olfactory memory from the relative times spent at different odour sites, which the animals must seek and find (Matsumoto and Mizunami, 2002), or the occurrence of a maxillary palpi extension response (MER), after either appetitive or aversive conditioning (Matsumoto et al., 2015). Casual observations of a cricket's immediate response to a conditioned odour reveal that the MER is only part of a more complex searching behaviour, involving tortuous movement in the vicinity of the conditioned stimulus, while extending the palps and waving the antennae, often interposed by characteristic bobbing movements of the head. Accordingly, we applied binary logistic regression to evaluate multiple behavioural components of this searching behaviour, which was captured by automated video-tracking and manual event scoring functions of commercial software (EthoVision). Binary logistic regression is a means of quantifying the unique contribution of each individual member of a group of independent variables to a binary outcome, in order to identify the strongest linear combination of variables with the greatest probability of detecting the observed outcome. See for example Bewick et al. (2005) on predicting health risks factors for death or survival and Stoltzfus (2011); Osborne (2012), for brief mathematical primers. In insects, the method has been exploited to characterise the different behavioural states of gregarious and solitary living locusts to understand mechanisms and causes of phase change (Roessingh et al., 1993; Simpson et al., 2001; Anstey et al., 2009; Gray et al., 2009; Cullen et al., 2012; Ott et al., 2012; Rogers et al., 2014), but not as far as we know to quantify learning and memory. In adherence to the general principles employed in these earlier studies on locusts, and using commercial software, we analysed the behavioural responses of a cohort of 50 crickets to a conditioned odour, and of another cohort of 50 towards a non-conditioned odour. Basically, we employed a standard, forward-stepwise procedure to select and test different behavioural response variables for inclusion in a mathematical model that can best discriminate between the two cohorts. The model was then used to calculate a probability for each individual cricket that it exhibited a conditioned response to an odour (*P*_resp_). Several potential models, with different combinations of variables, were constructed from the same data set and compared to determine the strongest. Using additional animal cohorts, we then tested the model's efficacy to differentiate the behavioural responses to an odour (conditioning stimulus, CS) that was paired with a sucrose reward as an unconditioned stimulus (CS + US-paired), compared to the responses of animals that received US-only, CS-only or unpaired presentations of the CS and US. Finally, we employed the model to measure individual *P*_resp_ values for crickets in a differential appetitive olfactory conditioning paradigm, and from this individual learning scores. We hypothesise that our multi-factorial analysis will prove superior to a single metric for estimating individual learning capacity, and test this by comparing model scores with estimates based on the characteristic head-bobbing response alone, which is itself a key component of the model.

## Materials and Methods

### Animals and Ethical Note

Mature, adult male crickets (*Gryllus bimaculatus*, De Geer) were reared under standard conditions. Some 30–40 individuals of both sexes were kept in perspex containers at the housing facility of the Leipzig University (22–24°C, relative humidity 40–60%, 12 h:12 h light-dark cycle) and fed regularly on protein flakes (Tetra GmbH, Melle, Germany) and fresh carrots, with water given *ad libitum*. Prior to the experiments the animals were isolated in glass jars for 48 h, supplied with protein flakes and deprived of water for the last 24 h. The experiments were performed at room temperature (20–24°C) after midday. All procedures conformed to the Principles of Laboratory Animal Care and German Law on Protection of Animals (*Deutsches Tierschutzgesetz*). The animals were kept in a separate breeding box after the experiments until the end of their natural lives. The analysis is based on 264 animals, each of which participated only once in one analysis.

### Odour Stimulation and Sucrose Reward

Two odours, often used in other studies (e.g., Scherer et al., 2003), served as conditioning stimuli (CS), 1-octanol (OCT; Merck KGaA, Darmstadt, Germany, applied pure) and amyl acetate (AM; Sigma-Aldrich, St. Louis, USA, diluted 1:10 in paraffin oil). At the used concentrations, experimentally naive animals showed no preference for either odour (three of the variables listed below were analysed, *time to move, total time moving* and *bobbing*; Mann-Whitney *U*-test s: *p* > 0.05 all cases, *N* = 50 each group, see also results). While working under a conventional fume hood, a filter paper (1 × 1 cm) was soaked with 5 μl odour solution using a micropipette and transferred inside a 10 ml syringe just in front of the tip. The filter paper was renewed after five odour applications. To ensure a greater odour volume signal, a plastic funnel (diameter: 15 mm) was fashioned from a disposable pipette and the thinner end (diameter: 7 mm) fixed to the syringe. Non-specific responses, due for example to sudden movements of the experimenter, were further minimised by positioning the syringe in the immediate vicinity of the animal approximately 30 s before odour application. With the funnel manually positioned circa 2 cm above the animal's antennae, odour was applied over the antenna by depressing the plunger with a smooth steady motion within 1.5 s when the animal was stationary on the floor of the arena. Experiments were conducted in a room with a ventilation system, and an additional fan was used to clear the air from the arena between successive odour applications and between experiments with different animals. The method of odour application was the same for the training and recall tests explained below and for all experiments.

In specific cases (see below), odour stimulation (CS) was followed by a sucrose reward as the unconditioned stimulus (US; time when applied differed as given below). For this, a drop of sucrose solution (7–10 μl, 1 M, Nordzucker AG, Braunschweig, Germany) formed on the tip of a 20 μl pipette was manually positioned in front of the animal's palps and the animal allowed to drink from this *ad libitum*.

### Setup and Video-Tracking

All experimental observations were performed in a recording chamber (80 × 60 cm and 79 cm high) lined on three sides with reflective card and illuminated by LED light panels (NL480, Neewer, Luo hu district, Shenzhen, Guangdong, China) arranged to minimise shadows cast from the animal. Individual animals were carefully transferred from their glass jars to arenas fashioned from an acrylic glass cylinder (diameter: 15 cm, height: 5 cm) placed upon a floor of cardboard covered with white paper that was exchanged for every animal. Each animal was marked with a dot of yellow acrylic paint (C. Kreul GmbH and Co. KG, Hallerndorf, Germany) on the head or pronotum for video tracking purposes. After allowing the animal to acclimatise for 20 min, the arena was transferred into the recording chamber.

**Figure 1:**
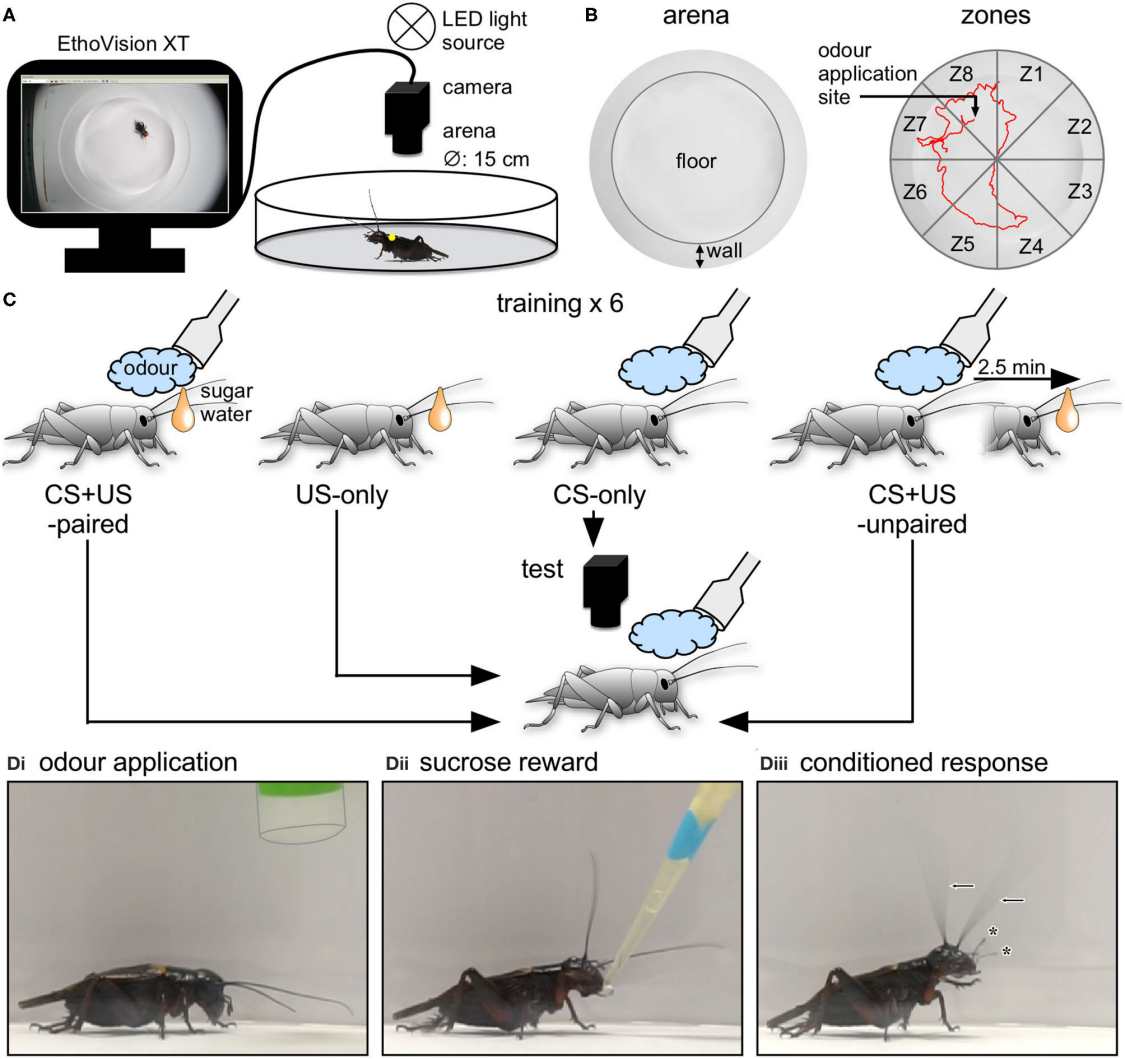
Experimental procedure. **(A)** Video-tracking setup. After marking, each cricket was placed in a circular arena and its motor response to a stimulus was filmed and digitally tracked using a digital video camera connected to personal computer running tracking software (EthoVision XT) for storage and analysis. **(B)** Left side: Top view of the arena showing the floor and side. Right side: Top view of the arena showing the eight virtually partitioned zones (Z1–Z8), used also for defining the stimulus site. The red line shows an exemplary 2 min track of an animal's movements in the arena. **(C)** Single odour conditioning paradigm employed to create two binary responding groups (US-only and CS + US-paired) used to build and test the model and two external control groups (CS-only; CS + US-unpaired). Individual crickets were trained (6*x*; inter-training-interval = 5 min) and were either given odour alone (CS-only, blue cloud), fed on sugar water (US-only, orange drop) or rewarded with a sugar water droplet directly after the presentation of the odour (CS + US-paired). CS + US-unpaired animals were presented with odour and reward with an interposing pause of 2.5 min. After 30 min the crickets were presented with the odour and simultaneously tracked with the video-tracking software for 2 min. **(D)** Photos showing examples of a cricket's responses to the initial application of an odour (pipette outlined), **(Di)** subsequent reward during training **(Dii)** and the conditioned odour response at the recall test, 30 min after training. **(Diii)** Note elevated head, waving antennae (arrows) and extended palps (asterisks).

Each animal's behaviour was filmed from above with a digital video camera (Basler acA1920-155uc, Ahrensburg, Germany, 60 frames/s; Figure 1A), then stored and analysed using commercial video-tracking software (EthoVision XT14, Noldus, Wageningen, Netherlands) running on a computer (Dell Precision 3620, Round Rock, Texas, USA) with Windows system software (10, Microsoft, Redmond, Washington, USA).

Regions of the arena (zones) were defined using EthoVision, with the tracking area (including the wall and floor) virtually partitioned into eight equally sized arcs used later to define where a stimulus was applied to the animal (Figure 1B). The yellow marker on each cricket was detected using the live, colour-marker tracking routine of EthoVision and tracking was based on an X/Y coordinate system calibrated in cm. To reduce jitter from ventilation movements and similar, a minimum distance moved filter was set to 0.05 cm, below which no movements were registered.

Before starting the experiment, the animal was allowed to acclimatise for 5 min in the setup. Odour was then applied to the animal as soon as it was stationary, while at the same time pressing the command to start recording. Recordings started 1 s after the command to give time for the system to detect the marker and allow the pipette to be removed from view. Recordings ended automatically after 2 min.

### Behavioural Response Variables

EthoVision offers numerous tracking values to evaluate movement. We selected eight variables judged to be biologically feasible indicators of a conditioned odour response (Table 1). The raw data for seven were provided automatically by EthoVision as a list of movement values for each 1/60 s frame over 2 min for each individual, and one parameter was measured manually (*bobbing*). All data were transferred to Excel (version 16.30, Microsoft, Redmond, Washington, USA) for subsequent calculations and minor editing given explicitly below:

**Table 1:** Statistical data for response variables.

**Variable**	**US-only median (IQR)**	**CS + US-paired median (IQR)**	***p* (U)**
*Time to move*, s	25.5 (10.3–86.7)	1.7 (0.9–4.7)	** <0.0001** (266)
*Initial distance*, cm	0.3 (0.1–0.6)	0.2 (0.2–0.4)	0.264 (1,087)
*Total distance*, cm	10.7 (2.1–25.4)	52.2 (29.4–99.1)	** <0.0001** (322)
*Total time moving*, s	2.9 (0.6–6.4)	14.1 (8.5–23.6)	** <0.0001** (299)
*Time moving in odour zone*, s	0.9 (0.3–1.5)	4.1 (1.9–6.4)	** <0.0001** (296)
*Time moving in odour area*, s	2 (0.6–3.3)	8.4 (3.8–14)	** <0.0001** (261)
*Velocity*, cm/s	3.7 (3.4–4)	3.9 (3.7–4.1)	**0.045** (959)
*Bobbing*, s	0 (0–0)	10.4 (2.3–27.1)	** <0.0001** (308)

*Median and interquartile range (IQR) are given (N = 50 each); p and U values are given from the Mann-Whitney U-test (boldface when significant)*.

*Time to move* (s): Time until a movement sequence began after odour application. The videos were manually checked to exclude data for any sudden and short movements (e.g., body jerks) that were not the beginning of a clear movement sequence and the value taken when a clear movement began. Individuals that did not show movement during the 2 min recording session were allocated the maximum value of 2 min.

*Initial distance* (cm): Sum of the distance moved in each time frame (1/60 s) for the first 4 s after the start of movement.

*Total distance* (cm): The total distance moved during the entire 2 min recording.

*Total time moving* (s): Total time spent moving during the entire 2 min recording.

*Time moving in odour zone* (s): Time moving in the zone where odour was applied.

*Time moving in odour area* (s): Time moving in the odour-and 2 adjacent zones.

*Velocity* (cm/s): Mean velocity for time frames when the animal was moving.

*Bobbing* (s): The cumulative time spent showing head-bobbing behaviour. This was often seen in response to a rewarded odour (see results for details) and was measured using the manual scoring function in EthoVision, and blinded regarding each animal's group identity and treatment.

### Calculating a Conditioned Odour Response Probability

Binary logistic regression analysis (review: Bewick et al., 2005; mathematical primers: Stoltzfus, 2011; Osborne, 2012), was applied to construct a mathematical model for calculating a probability for each individual animal of it exhibiting a conditioned response to an odour (*P*_resp_). We adhered to the same basic principles applied in earlier studies on locusts (Gray et al., 2009; Cullen et al., 2010, 2012; Rogers et al., 2014; Ott, 2018). We constructed several models, which we later compared to determine their relative accuracy. Each model was based on the behavioural responses of the same two, binary groups of animals to a single odour in an absolute conditioning paradigm. For this, we first recorded each individual cricket's response to presentation of one of the two odours before training to check for differences in the naive response (US-only naive, given in results). After a 5 min pause, individuals of one group, CS + US-paired (*N* = 75), received the same odour as before and were then given sucrose water to drink immediately afterwards as a reward (<2 s). Individuals of the second group, US-only animals (*N* = 75), received sucrose alone to preclude behavioural differences due to satiation level. These training procedures were repeated six times with the aim to maximise the behavioural differences between the two groups. As in other studies (e.g., Behrends and Scheiner, 2012), animals were discarded from further analysis if they did not drink from the sucrose solution at any time during training or when they died within the two days following the experiment. Training was followed after a 30 min pause by a recall test, in which each individual's response to a single presentation of odour was recorded (Figure 1C).

A random number generator (Excel: Mersenne Twister-Algorithm) was used to select 50 individuals from each binary group to build the model. The remaining 25 from each group (US-only, CS + US-paired) were used to test the model. For control purposes, two additional groups were generated: individuals of one group received only odour at each of the six training trials spaced at 5 min intervals (CS-only, *N* = 25), and the other received odour followed by the reward after a 2.5 min pause, followed by an additional 2.5 min pause before starting the next of six training sessions (CS + US-unpaired, *N* = 25). Behavioural variables from video tracks of the 50 US-only and 50 CS + US-paired animals were entered into a forward stepwise conditional binary logistic regression analysis program (SPSS Statistics, Version 25, IBM, Armonk, New York, USA) running on a Macintosh computer (Apple Computers, Cupertino, CA, USA). The probability levels for stepwise entry and removal of a variable into a model were set to 0.05 and 0.1, respectively. The model provides a means of calculating a single metric giving a probability of an individual that it exhibited a conditioned response to an odour, *P*_resp_, given by the logistic algorithm:


$$\begin{array}{l} P_{\text{resp}}=\;\frac{e^{\eta }}{\left(1+e^{\eta }\right)} \end{array}$$

whereby η is the sum of an intercept β_0_ plus measures of each behavioural variable (*x*) found to be a strong predictor of a conditioned odour response, weighted by a coefficient β for each variable as given by the regression equation:


$$\begin{array}{l} \text{and }\eta =\;\beta_{0}+\beta_{1}x_{1}+\beta_{2}x_{2}+\;\ldots \;+\;\beta_{k}x_{k} \end{array}$$

The ideal model should correctly discriminate between all individuals in the two groups, whereby individuals showing a *P*_resp_ greater than the cut off value of 0.5 are allocated by the model to the CS + US group, and individuals scoring < 0.5 are allocated to the US-only group. Comparing these data with the actual treatments then gives the degree of correct assignment by the model, which we give as a percentage (Table 2). We describe and compare four models constructed from different variables. The Wald statistic was applied to test the significance of contribution of each variable in a model and the Hosmer-Lemeshow test to evaluate the model's goodness-of-fit (Hosmer and Lemeshow, 2000). The latter yields the probability that the model's allocation of individuals to the binary groups is significantly different to their actual distribution, i.e., a good fit should have a *p* > 0.05. The Akaike information criterion for small sample sizes (AICc; Akaike, 1974) was calculated (Prism 8, GraphPad Software Inc., La Jolla; CA, USA) for each model to estimate their relative prediction quality, whereby a lower AICc score indicates a superior model. The area under the receiver operating characteristic curve (AUC under ROC, Hosmer and Lemeshow, 2000; Austin and Steyerberg, 2012) was used to measure the model's ability to discriminate between animals of the US-only and CS + US-paired group. An AUC under ROC of ≥ 0.9 is considered as outstanding discrimination. Finally, the discriminating power of the most favourable model was tested using variables from tracked recordings of the remaining animals of the CS + US-paired and US-only groups, as well as with the two additional control groups that received CS-only and CS + US unpaired during training.

**Table 2:** Comparison of binary logistic regression models evaluated for calculating the probability of an animal showing a conditioned odour response (*P*_resp_).

		**Coef**		**Wald stat**.		**Assignment %**					
***Model***	**Variable**	**ß**	**SE**	**Chi^**2**^**	***p***	**Avg**.	**US-only**	**CS + USp**	**HL*-p***	**AICc**	**AUC**
***-1***	*Time to move* *Total time moving* *Bobbing* *Intercept*	−0.156 0.152 0.985 −0.973	0.061 0.067 0.377 0.818	6.523 5.166 6.838 1.417	**0.011** **0.023** **0.009** 0.234	93 (90)	96 (92)	90 (88)	0.988	45.35	0.975 (0.960)
***-2***	*Time to move* *t-mov. odour area* *Bobbing* *Intercept*	−0.148 0.199 0.933 −0.458	0.059 0.119 0.363 0.759	6.351 2.813 6.627 0.363	**0.012** 0.094 **0.010** 0.547	91 (86)	96 (84)	86 (88)	0.988	48.9	0.970 (0.955)
***-3***	*Time to move* *Total time moving* *Velocity* *Bobbing* *Intercept*	0.156 0.205 1.598 0.956 4.826	0.064 0.084 1.406 0.375 5.151	5.880 6.012 1.292 6.507 0.878	**0.015** **0.014** 0.256 **0.011** 0.349	94 (90)	98 (92)	90 (88)	0.999	46.11	0.976 (0.938)
***-4***	*Time to move* *Initial distance* *t-mov. odour area* *Intercept*	0.064 1.529 0.340 0.018	0.028 0.745 0.107 0.755	5.053 4.217 10.00 0.001	**0.025** **0.040** **0.002** 0.981	87 (82)	88 (84)	86 (80)	0.114	74.68	0.936 (0.904)

*Model-1: the most parsimonious; Model-2: suggested by SPSS; Model-3: has an additional, 4th, variable; Model-4: excludes bobbing. The table gives the variables retained in a model plus its regression intercept for each of the coefficients (Coef ß), the standard error (SE) and Wald statistic (Chi^2^ and p, boldface when variable contribution is significant). The percentage of animals assigned correctly to the US-only or CS + US-paired (CS + USp) group (N = 50 each) and the average (Avg.) is given for animals included in the model, and in parentheses for out-model test animals (N = 25 each). The Hosmer-Lemeshow test gave the goodness-of-fit probability (HL-p) for each model. We also give for each model the Akaike information criteria score (AICc, smaller values indicate a superior model) and the area under the receiver operating characteristic curve (AUC, in parentheses for out-model test animals; values over 0.9 indicate outstanding discrimination)*.

### Differential Olfactory Conditioning

The most parsimonious model was applied to evaluate individual learning scores in a differential appetitive olfactory conditioning paradigm using the same odours as for constructing the model with a fresh cohort of 64 animals (see results). In this paradigm, each animal was first presented with each odour in succession (2 min each, with a 3 min pause) and after a 10 min pause, this was followed by training during which the animal was again given each odour alternately, whereby one odour was rewarded with sugar water (CS^+^) and the other not (CS°). Training was repeated three times (at 5 min intervals), rather than six times as for constructing the binary logistic regression model, in order to obtain a broader spectrum of odour responses at the recall test 30 min later. During the latter, each odour was presented only once with a pause between (3 min). The sequence in which each odour was presented and rewarded was varied systematically to give eight different regimes (*N* = 8 each; see results). Video-tracks of each animal at the recall test were analysed to obtain *P*_resp_ values to the rewarded (CS^+^) and the unrewarded odour (CS°) for each individual and from the difference the individual learning indexes (*LI* = *P_resp_* CS^+^ – *P_resp_* CS°). Since the *LI* is based on probabilities, we assume that a difference between two *LI*s > 0.05 is significant.

### Additional Statistical Tests

Additional statistical tests were performed with Prism 8. The Shapiro-Wilk test revealed that our data sets were not normally distributed. Accordingly, we give for each variable the median, interquartile range (IQR) and the 10 + 90 percentiles and applied non-parametric statistical tests: the Mann-Whitney *U*-test to test for significant difference between unpaired data sets, and the Wilcoxon signed rank test for paired data sets. A one-way ANOVA on ranks (Kruskal-Wallace test) was applied to determine if there are statistically significant differences in learning scores between the eight different regimes of the differential conditioning paradigm. The significance level alpha was set to *p* < 0.05, excepting the few occasions when the same data set was used for two separate statistical tests, in which case we applied the Bonferroni correction to alpha = 0.025 for multiple comparisons (indicated explicitly in text). In one instance we calculated a bimodality coefficient for a data set to test for a bimodal distribution according to Pfister et al. (2013).

## Results

### Behavioural Responses to Odour

**Figure 2:**
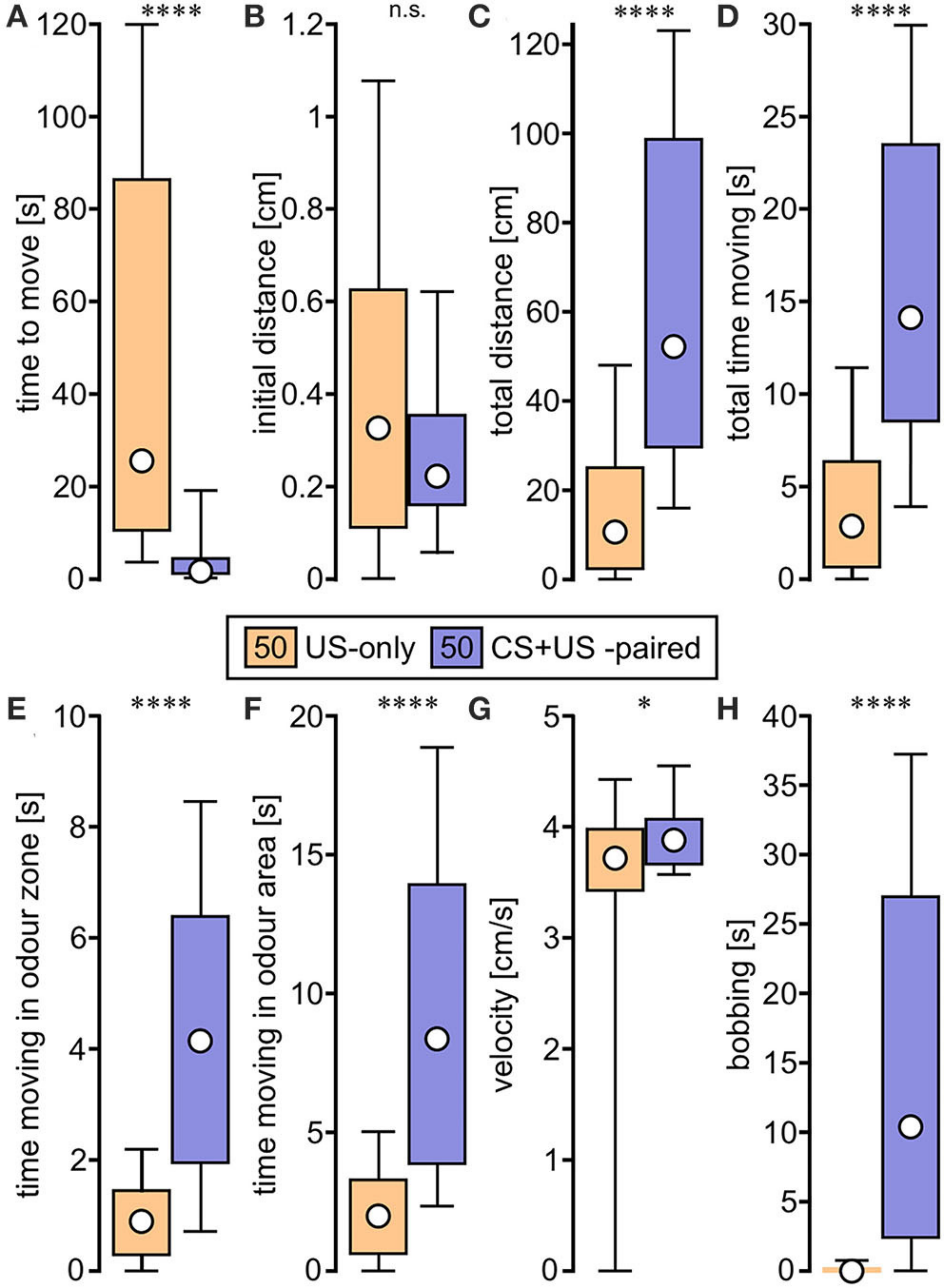
Box plots of variables extracted from video tracked odour responses and used for binary regression analysis. The animals were trained in the single odour (absolute) conditioning paradigm (Figure 1) and received either US-only (orange) or CS + US-paired (purple; circle: median; box: interquartile range; whiskers: 10th and 90th percentile; *N* = 50 for each group). **(A)** *Time to move*. **(B)** *Initial distance*. **(C)** *Total distance*. **(D)** *Total time moving*. **(E)** *Time moving in odour zone*. **(F)** *Time moving in odour area*. **(G)** *Velocity*. **(H)** *Bobbing*. Asterisks indicate significant differences: Mann-Whitney *U*-test; *p < 0.05; ****p < 0.0001; n.s., not significant.

We first evaluated odour responses of crickets after single odour conditioning (cf. Figure 1). In general, individuals previously trained to an odour by pairing it with a sucrose reward (CS + US-paired) subsequently responded to that odour by moving around the arena, while waving their antennae and bobbing their heads. This apparent searching behaviour for the sucrose reward was formally quantified by comparing the video-tracked odour responses of trained individuals, that received the odour followed by sucrose reward with those individuals that received the sucrose reward only (CS + US-paired and US-only, respectively, *N* = 50 each; Figure 2, see Table 1 for statistical details).

Regarding *time to move*, CS + US-paired trained crickets began moving around the arena within a few seconds after odour stimulation at the memory recall test, whereas US-only crickets started moving significantly later, or not at all during the 2 min recording session (*p* < 0.0001, Figure 2A). Although the *initial distance* moved in the first 4 s of movement did not differ between the two groups (*p* = 0.264, Figure 2B), the *total distance* covered during the 2 min recording session was significantly longer for the CS + US-paired group compared to US-only (*p* < 0.0001, Figure 2C). Similarly, the durations recorded for *total time moving, time moving in odour zone* and *time moving in odour area* were all significantly greater for the CS + US-paired group (*p* < 0.0001 in all three cases, Figures 2D–F). During movement, the *velocity* for CS + US-paired crickets was faster than the US-only group, but this was only just significant (*p* = 0.045, Figure 2G). Finally, clear *bobbing* behaviour was shown by the majority of CS + US-paired crickets (78%) and for over half of these animals this lasted more than 10 s. The *bobbing* duration for US-only crickets was significantly less (*p* < 0.0001, Figure 2H) and was exhibited by only 10% of the animals, for maximally 3.8 s.

### Building and Selecting a Binary Logistic Regression Model

Based on the data from the two groups (US-only and CS + US-paired, *N* = 50 each), binary logistic regression analysis with the forward conditional procedure was employed to generate a mathematical model to calculate the probability that an animal exhibited a conditioned response to an odour. The most parsimonious model (*Model-1*) was generated after testing and gaining information from a variety of alternatives, three of which we describe here for comparative purposes (*Models 2, 3 and 4*; detailed statistics in Table 2).

*Model-1* retained the three variables *time to move, total time moving* and *bobbing*. The Wald statistic for each variable was significant (*p* < 0.05), indicating that all variables made a significant contribution to the model. In all, 93% of the animals were allocated to the correct group by the model (US-only 96%, CS + US-paired 90%), and the Hosmer-Lemeshow goodness-of-fit test yielded a *p*-value greater than 0.05 (*p* = 0.988), indicating that the model's allocation of individuals to the CS + US-paired or US-only groups is not significantly different to their actual classification. Mathematical modifications to data sets brought no improvements to the model. For example, *log*_*n*_-transformation of the data for *bobbing* generated a less superior model (89% correctly allocated, Hosmer-Lemeshow *p* = 0.71; AICc scores *Model-1*: unmodified 45.35, modified 51.57).

*Model-2* was suggested by the statistics program SPSS and based on the variables *time to move, time moving in odour area*, and *bobbing*. Although the Hosmer-Lemeshow test indicated equal goodness-of-fit (*p* = 0.988), *Model-2* was rejected for the following reasons. First, it assigned fewer animals than *Model-1* to their correct groups (average 91%, US-only 96%, CS + US-paired 86%). Second, the *p-*value (*p* = 0.094) from Wald statistic for *time moving in odour area* indicates that this variable does not contribute significantly to the model. Third, the AICc score (48.9) was higher than for *Model-1*.

*Model-3* is the best we could construct using four variables and included the same three variables as *Model-1 (time to move, total time moving, bobbing)*, plus *velocity*. *Model-3* assigned more animals into their correct groups than *Model-1* (94%, Hosmer-Lemeshow, *p* = 0.999), but we rejected this model on the grounds that the contribution of *velocity* is insignificant (Wald statistic: *p* = 0.256) and the AICc-score was higher than for *Model-1*.

*Model-4* represents our best attempt to generate an automatic model excluding *bobbing*, which we could only measure manually. This model included three variables: *time to move, initial distance* and *time moving in odour area*, and was the least strongest (Hosmer-Lemeshow: *p* = 0.114; AICc score: 74.68). Even so, all three variables made a significant contribution to the model, which classified 87% of the animals correctly.

All models have an AUC under ROC of over 0.936 indicating that all models show an outstanding discrimination.

### Odour Response Probabilities (*P*_resp_) With *Model-1*

The coefficients ß for each variable in *Model-1* were then used to calculate a probability of exhibiting a conditioned odour response for each animal included in the model from:


$$\begin{array}{l} P_{\text{resp}}=\;\frac{e^{\eta }}{\left(1+e^{\eta }\right)} \end{array}$$

whereby,


$$\begin{array}{l} \eta =\left(-0.973\right)+\left(0.152*total\;time\;moving\right)-\\ \;\left(0.156*time\;to\;move\right)+\;\left(0.985*bobbing\right). \end{array}$$

**Figure 3:**
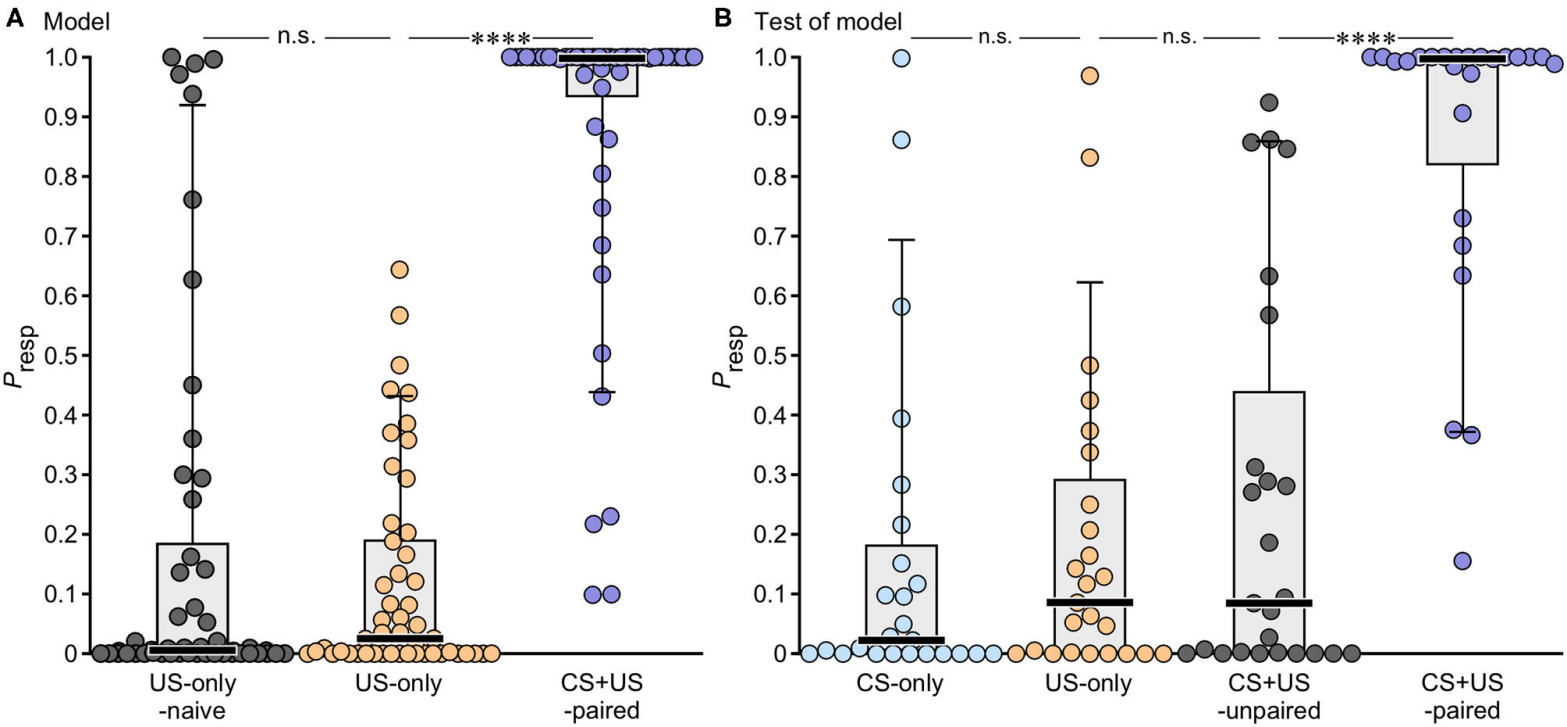
Box plots giving the individual probabilities of showing a conditioned odour response (*P*_resp_) as derived from binary logistic regression modelling. **(A)** *P*_resp_ to odour for animals used to construct the model (US-only, CS + US-paired, *N* = 50 for each plot), together with data for US-only animals before training when experimentally naive (naive US-only, *N* = 50). **(B)** *P*_resp_ data for animals used to test the model (*N* = 25 for each plot). Prior to testing they received either CS-only (blue), US-only (orange), CS + US-unpaired (grey) or CS + US-paired (purple). Circles: individual values; bar: median; box: interquartile range; whiskers: 10th and 90th percentiles. Asterisks indicate significant differences: Wilcoxon signed rank test (naive US-only), Mann-Whitney *U*-test (all other groups); ****p < 0.0001; n.s., not significant.

A *P*_resp_ of 0 indicates that an animal did not respond to the odour whereas a *P*_resp_ of 1.0 means an absolutely certain response. The resultant *P*_resp_ values for the data from animals used to build the model are illustrated in Figure 3A with statistical details in Table 3. Animals that received US-only showed low *P*_resp_ values (median: 0.025), with only two individuals exhibiting a *P*_resp_ greater than the cut-off at 0.5. For comparative purposes we also show data for experimentally naive US-only animals before training. This revealed that sucrose application alone during training had no significant effect on the *P*_resp_ value (Wilcoxon signed rank test: *p* = 0.835). Compared to US-only, the *P*_resp_ for CS + US-paired was significantly greater (median: 1.0, *p* < 0.0001), with five animals having a *P*_resp_ below the cut-off at 0.5.

**Table 3:** *P*_resp_ values for all test groups of animals used to construct (in-model) and test (out-model) the binary logistic regression *Model-1*, and also for the differential conditioning paradigm (*Model-1, bobbing*).

**Test group**		**Median**	**IQR**	***N***	***p***	***U/W***
In-model	Naive US-only	0.005	0–0.186	50	0.835	*W*-42
	US-only	0.025	0–0.192	50	** <0.0001**	*U* 63
	CS + US-paired	1	0.932–1	50		
Out-model	CS-only	0.022	0–0.184	25	0.295	*U* 258
	US-only	0.086	0–0.294	25	0.758	*U* 296
	CS +US-unpaired	0.085	0–0.44	25	** <0.0001**	*U* 36
	CS + US-paired	0.997	0.818–1	25		
Differential conditioning	CS°	0.204	0–0.708	64	** <0.0001**	*W* 1716
*Model-1*	CS^+^	0.983	0.654–1	64		
Differential conditioning	CS°	0	0–0.019	64	** <0.0001**	*W* 1130
*Bobbing*	CS^+^	0.054	0–0.216	64		

*Values for p are boldface where significant, U is from Mann-Whitney U-tests, and W from Wilcoxon signed rank tests, alpha is set to 0.025 due to two comparisons using the same data set*.

The predictive power of *Model-1* was also evaluated with out-model data from groups of additional test-animals not used for model construction (*N* = 25 for each group, Figure 3B, Table 3). On average, *Model-1* allocated 90% of the out-model animals to their correct groups (US-only 92%, CS + US-paired 88%), and in this respect was superior to *Models-2* and *4*, and equalled *Model-3* (see Table 2). Out-model animals that received US-only showed low *P*_resp_ values (median: 0.086), with only two animals showing a response greater than the cut-off value of 0.5. CS-only animals, which received odour only during training, had an equally low *P*_resp_ score (median: 0.022, *p* = 0.295), with only three animals showing a *P*_resp_ greater than 0.5. Animals that received the odour and the reward unpaired also showed low *P*_resp_ values compared to US-only (CS + US-unpaired, median: 0.085, *p* = 0.758), with six animals showing a *P*_resp_ greater than 0.5. In contrast, out-model CS + US-paired animals showed significantly higher *P*_resp_ values than CS + US-unpaired (median: 0.997, *p* < 0.0001) with only three animals scoring under 0.5.

### Applying the Binary Regression Model to Measure Learning

We next applied our binary logistic regression model to measure learning in a differential appetitive olfactory conditioning paradigm, in which two odours were presented during training, but only one rewarded. The eight different regimes in which the rewarded odour and presentation sequence was varied systematically are depicted in Table 4. For each of the eight different animals in each regime, we used *Model-1* to equate the *P*_resp_ values for the rewarded odour and non-rewarded odour at the recall test (CS^+^ and CS°, respectively) and from this a learning index (*LI*) for each individual animal. The *LI* for each of the eight regimes did not differ significantly from each other (Kruskal-Wallace test, *p* = 0.334, *H* = 7.985, not illustrated). Similarly, it made no significant difference to the *LI* which of the two odours (AM and OCT) was presented first at training, or at recall, or which odour was rewarded. We also found no influence depending on whether the rewarded odour (CS^+^) was presented first or second during training, or at the subsequent recall test (Table 5). Accordingly, we pooled the data for all 64 animals for a detailed analysis.

**Table 4:** Differential olfactory conditioning paradigm.

	**Naive**				**Training 3** ***x***				**Recall test**		
**Seq**.	**1st**	**5 min**	**2nd**	**5 min**	**1st**	**5 min**	**2nd**	**30 min**	**1st**	**5 min**	**2nd**
1	AM		OCT		**AM**		OCT		AM		OCT
2	AM		OCT		**OCT**		AM		OCT		AM
3	AM		OCT		OCT		**AM**		AM		OCT
4	AM		OCT		AM		**OCT**		OCT		AM
5	OCT		AM		**OCT**		AM		AM		OCT
6	OCT		AM		**AM**		OCT		OCT		AM
7	OCT		AM		AM		**OCT**		AM		OCT
8	OCT		AM		OCT		**AM**		OCT		AM

*Animals were presented with two different odours in succession (amyl acetate, AM and 1-octanol, OCT), each once before training (naive), each three times in succession during training, whereby one odour (bold and underlined) was paired with a reward. Finally, each odour was presented once more at the recall test which was filmed. Pauses between applications are indicated. The actual sequence of when and which odour was applied and rewarded was varied to give eight different permutations (Seq. 1–8, eight animals each)*.

**Table 5:** Data from tests for differences in learning index (*LI*) based on the *P*_resp_ values for the eight permutations of the differential olfactory conditioning paradigm (Table 4).

**Odour…**		***LI* mean**	**Median/IQR**	**10−90%**	***p* (U)**
…given 1st in training	AM	0.319	0.096 (0.003–0.690)	−0.013–0.995	0.132 (399)
	OCT	0.513	0.626 (0.217–0.949)	−0.228–0.995	
…given 1st at recall	AM	0.436	0.436 (0.013–0.906)	−0.216–0.998	0.526 (464)
	OCT	0.396	0.365 (0.001–0.858)	−0.084–0.990	
…rewarded in training	AM	0.435	0.479 (0.010–0.847)	−0.013–0.985	0.826 (495)
	OCT	0.397	0.314 (0.002–0.972)	−0.228–0.997	
…rewarded, given in training	1st	0.355	0.238 (0.001–0.906)	−0.228–0.987	0.221 (420)
	2nd	0.477	0.526 (0.058–0.890)	0.000–0.998	
…rewarded, given at recall	1st	0.324	0.096 (0.000–0.837)	−0.302–0.998	0.132 (399)
	2nd	0.508	0.526 (0.208–0.949)	0.001–0.991	
	All data	0.416	0.395 (0.005–0.890)	−0.332–0.999	<0.0001

*For each of the five tests performed, the entire data set for 64 animals was divided into two, but in different ways to generate five pairs of data sets, which differed in only one aspect regarding either the identity or presentation sequence of the rewarded odour during training and at the recall test. Significant differences were not found (Mann-Whitney U-test, p > 0.05 in all cases). The last column gives data for the entire group of 64 animals (Wilcoxon signed rank test with hypothetical value = 0; p <0.0001; W = 1,716)*.

**Figure 4:**
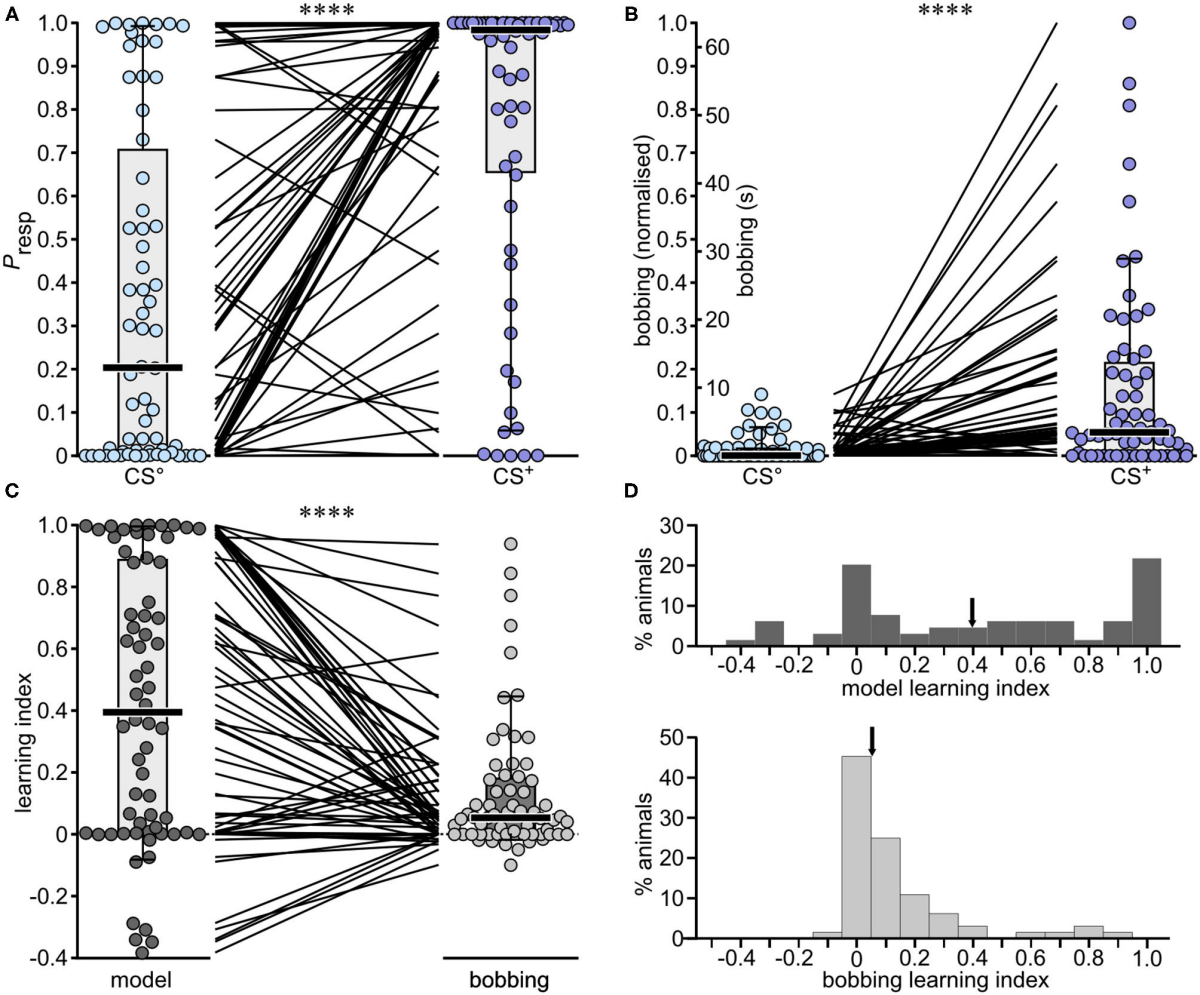
Performances of individual crickets in the differential olfactory conditioning paradigm: A comparison of the binary regression *Model-1* with *bobbing*. **(A)** Boxplots giving *P*_resp_ values derived from the binary regression model for the non-rewarded and rewarded odour (CS° and blue circles, respectively CS^+^ and purple circles; bars: median; boxes: interquartile range; whiskers: 10th and 90th percentiles, *N* = 64). The interposing lines between plots indicate the difference in response for each individual. **(B)** As for **(A)**, but for *bobbing*, for which the duration was normalised for the entire dataset [CS° and CS^+^: 0 = lowest value; 1 = highest; the original data scale *bobbing* (s) is also depicted for comparison]. **(C)** Comparison of the learning indexes given by *Model-1* and from normalised *bobbing* alone [CS^+^ minus CS° values from **(A,B)** respectively]. Asterisks in **(A-C)** indicate significant differences (Wilcoxon signed rank test; ****p < 0.0001). **(D)** Histograms showing the distribution of the model learning index (top) and the bobbing learning index (bottom). Arrows indicate the medians.

At the recall test, one third (33%) of the 64 animals showed a *P*_resp_ to the non-rewarded odour greater than the cut-off value 0.5 (CS° median: 0.204, Figure 4A, Table 3). Nonetheless, for the majority of animals the *P*_resp_ to the rewarded odour (CS^+^) was greater than to CS° (CS^+^ median: 0.983, significantly different to CS°, *p* < 0.0001), as indicated for each animal by adjoining lines in Figure 4A. The difference rendered a median *LI* of 0.395 (Figure 4C), with the majority (69%) scoring >0.05, indicating a significant appetitive learning effect, and 28% scoring > 0.8. Even so, 20% showed no significant learning (*LI* > −0.05, < 0.05), and 11% scored below −0.05, indicating learnt aversion to the rewarded stimulus.

### *Bobbing* As a Single Metric for Learning

Since *bobbing* appears to be a characteristic response to an appetitive olfactory signal in crickets, we checked whether this parameter is sufficient to evaluate learning and compared its efficacy to that of our binary logistic regression *Model-1* based on *bobbing* and two additional variables. For better comparison with the model data, we normalised the entire data set (CS° and CS^+^) so that the lowest value was 0 and the highest 1, but also indicate in the corresponding figure the non-normalised original values, which have the same relative distribution (Figure 4B).

The normalised *bobbing* score in response to the non-rewarded odour (CS°) was smaller than the cut-off value 0.5 (median: 0; Figure 4B) for all individuals in the group. The majority of animals also responded to the rewarded odour with higher scores (indicated by adjoining lines in Figure 4B). A comparison between the *LI*s given by *Model-1* and from *bobbing* alone revealed a significantly lower score for *bobbing* (median: *model-LI:* 0.395*, bobbing-LI:* 0.054, *p* < 0.0001, Figure 4C), with 53% of animals scoring greater than 0.05 but only 3% > 0.8, and 45% showing no appetitive learning (< 0.05 and > −0.05), and 2% aversion. The difference between the two *LI*s is also illustrated in the frequency histogram of Figure 4D. Whereas, the data for the *bobbing-LI* have a skewed distribution, with a maximum of 0, scores for the *model-LI* show two modes, at 0 and 1.0. For the latter, we calculated a coefficient of bimodality of 0.558, whereby significant bimodality is indicated by values greater than the critical value of 0.555 (see Pfister et al., 2013).

## Discussion

In this paper we employed binary logistic regression analysis of video-tracked behaviour to build a mathematical model for calculating the probability that a cricket exhibits a conditioned response to a previously trained odour (*P*_resp_). We found that the model can be used to compute learning scores for individual crickets in a differential appetitive olfactory learning paradigm, and confirmed our postulation that a multi-factorial model can evaluate the learning capacity of individuals more accurately than a single behavioural predictor.

Since we aimed to use a binary logistic regression model for estimates of a conditioned odour response in future studies, we first needed to build the model with data from two binary groups that differ as far as possible in their response to an odour (see Roessingh et al., 1993 on gregarious and solitarious locusts). For our model, we used absolute conditioning to compare the odour responses of 50 individuals that were previously trained to an odour (the conditioned stimulus, CS) paired with a sucrose reward (the unconditioned stimulus, US), to those 50 individuals that received the reward only (US-only). We opted for six training trials to maximise the difference in odour response between the two groups. In a comparable training regime, 80% of honey bees show the proboscis extension reflex (PER) as the conditioned response (Menzel et al., 2001), whereby more repetitions can lead to reduced US-related motivation due to increased satiation (Menzel et al., 2001; for Drosophila see also Krashes and Waddell, 2008). For this reason, we also chose CS + US-paired and US-only as our binary groups since the animals had balanced satiation levels, and subsequently controlled for effects of CS-only and CS + US-unpaired, where the US was applied 2.5 min after the CS. Contrary to some conditioning studies with crickets (Matsumoto and Mizunami, 2002), training and the recall test were performed in the same arena to minimise possible differences due to handling and changed surroundings. Furthermore, whereas many conditioning paradigms require the animal to locate an odour source, or visual stimulus in a Y-maze, or similar (e.g., Matsumoto and Mizunami, 2000; Watanabe et al., 2003; Giurfa, 2004; Dupuy et al., 2006), we applied the odour directly over the antennae, and video-tracked each animal's immediate response over the following 2 min. As a specific response to a conditioned odour, our crickets engage in characteristic searching behaviour in the near vicinity of the odour application site. To quantify this, we analysed eight movement variables, of which seven differed significantly in the CS + US-paired and US-only groups. On average, CS + US-paired trained crickets started moving earlier, moved further and slightly faster, and spent more time moving, especially in the vicinity of the odour stimulus, while engaging for a longer period in head-bobbing movements.

The results revealed that this experimental regime generated binary groups that could be distinguished with high accuracy by binary logistic regression analysis. Using forward-stepwise binary logistic regression analysis we constructed a variety of mathematical models that implemented various combinations of the eight behavioural variables to discriminate between the two binary groups (CS + US-paired, US-only). From several models tested, the most parsimonious and accurate model (*Model-1)* was based on only three variables: *time to move, total time moving* and *bobbing*. This model had an overall discrimination accuracy of 93%, which compares favourably with the averages of 89.5–90.7% given by several three-and four, or more, variable models that discriminate between the behaviour of gregarious and solitary locusts (see Cullen et al., 2012 for references). As applied in other studies (Gray et al., 2009; Cullen et al., 2012; Stettin, 2014; Ott, 2018), the superiority of our *Model-1*, compared to other models tested, was verified by the Hosmer-Lemeshow goodness-of-fit test (Hosmer and Lemeshow, 2000), the Akaike information criteria score (Akaike, 1974) and the area under the receiver operating characteristic curve (AUC under ROC, Hosmer and Lemeshow, 2000; Austin and Steyerberg, 2012). The *P*_resp_ values calculated from *Model-1* for all animals used to build the model were mostly around zero for the US-only group and 1.0 for the CS + US-paired group (see Table 3), and were thus clearly differentiated. Furthermore, similarly, low values for the US-only group before training when experimentally naive, revealed that the sucrose reward alone had no significant effect on the odour response. Similarly, *P*_resp_ values for out-model animals, that were not used for model construction, confirmed the accuracy of *Model-1* for calculating a conditioned odour response. Individuals of an additional out-group, given odour only during training (CS-only), had low *P*_resp_ values, confirming that multiple presentations of odour alone do not lead to high scores (see Matsumoto and Mizunami, 2002). Furthermore, out-group individuals receiving CS + US-unpaired during training had correspondingly low *P*_resp_ scores, confirming that high scores for the CS + US-paired group result from associative conditioning.

The viability of our binary logistic regression model for scoring learning in individual crickets was verified in a differential appetitive olfactory conditioning paradigm. For this, the *P*_resp_ to two different odours were compared, of which one was rewarded during training and the other not (CS^+^, CS°, respectively). We performed three training repetitions for differential conditioning, which has proven to be sufficient in other studies of crickets (Matsumoto and Mizunami, 2002) and other insects (Sandoz et al., 1995; Watanabe et al., 2003; Gerber et al., 2013). Application of the model revealed that the majority of crickets (69%) differentiated between the two odours at the recall test 30 min after training (Figure 4) by showing a higher *P*_resp_ to CS^+^ than to CS°. Notably, the *P*_resp_ scores to CS° were higher than obtained for CS-only in single odour conditioning (CS-only, Figure 3B). Considering the low response of crickets to the CS after training with CS + US-unpaired in control experiments (Figure 3B), it is unlikely that the relatively high *P*_resp_ to CS° in the differential conditioning paradigm is due to any difference in sensory experience during training. It is more likely that a high score arises because there were fewer training repetitions for the differential learning paradigm than for absolute conditioning, and the task was more complex, in that it required the animals to differentiate between two odours that are equally attractive to experimentally naive crickets. Honey bees, for example, tend to generalise the unrewarded odour in the first trials of a differential conditioning paradigm, especially when the odours are structurally similar (Smith and Menzel, 1989; Guerrieri et al., 2005; Lehmann et al., 2011).

Since we obtained odour response scores as a probability (*P*_resp_) for each individual cricket to both the conditioned and non-conditioned odour, we could calculate an appetitive learning index (*LI*) directly from the difference of the two measures. This differs to most procedures in invertebrates where a “preference-,” “performance-,” “discrimination-,” or “learning-” index is typically estimated from the net preference of a group of animals or as percentage of animals showing a specific response (Selcho et al., 2009; Lehmann et al., 2011; Apostolopoulou et al., 2013; Gerber et al., 2013; Nishijima and Maruyama, 2017), and only rarely for single animals (Drosophila larvae: Scherer et al., 2003; honey bees: Scheiner et al., 2020). Differential learning has been previously shown in crickets by comparing pre-and post-training preferences in an appetitive-aversive paradigm, in which an initially unattractive odour was rewarded and another initially attractive odour punished (Matsumoto and Mizunami, 2002). To our knowledge, however, we are the first to give individual learning scores, based on a multi-parameter semi-automated analysis of a conditioned response in a differential, appetitive paradigm. As in other studies (Scherer et al., 2003), we took the precaution to check for effects related to the identity and sequence of the odour presented in eight systematic variations of the procedure, but found none and hence pooled the data. In our paradigm, 69% of the crickets showed a significant learning effect (*LI* > 0.05) and were able to associate the correct odour with the reward. This percentage compares reasonably well with estimates of learning in crickets based on the maxillary palpi extension response (Matsumoto et al., 2015) and in honey bees based on the proboscis extension reflex (Lehmann et al., 2011): under reasonably comparable conditions, and after six training repetitions, around 80% respond to a conditioned odour and about 20–30% to an unconditioned odour. Direct comparisons are otherwise difficult due to methodological differences.

One of the main questions we posed was whether a multi-factor analysis is superior to a single behavioural metric for evaluating the behavioural response to a conditioning stimulus, as used in most learning paradigms (e.g., Bitterman et al., 1983; Matsumoto et al., 2015; Arican et al., 2020). We selected to analyse head-bobbing, since this appears to be a reasonably selective, immediate response to a conditioned odour, and is also the most weighted variable in our model, that is easily evaluated from video recordings. Although bobbing-behaviour has not previously been described in crickets, some of its components have been observed under various circumstances, e.g., antennal waving or fencing in response to mechanical stimulation (Balsam and Stevenson, 2020), during courtship (Adamo and Hoy, 1994) and aggression (Rillich and Stevenson, 2015), or palpal extension while raising the head in response to water on the antennae or a conditioned odour (Matsumoto et al., 2015). Although head-bobbing is a good indicator of a conditioned response, it is not absolutely specific. It also occurs when a feeder pipette with sugar water is removed before satiation, and in response to an attractive odour, though rarely when not rewarded. The same applies to most single behavioural measures of a conditioned response, such as palpal movements in crickets (Matsumoto et al., 2015), and the PER in honey bees (Bitterman et al., 1983). Due to this, experimentally naive individuals that respond spontaneously to the odour stimulus are often discarded from learning trials (see e.g., Behrends and Scheiner, 2012). We opted not to do this, and accept the risk of a lower average learning performance, as we wanted to see the entire spectrum of individual learning capacities in an unbiased random selection of laboratory animals.

A comparison of *P*_resp_ scores with bobbing scores shows that the binary regression model for analysing learning is superior. The individual learning scores for the model, were significantly greater than for the *bobbing-LI* (median: 0.395 and 0.054, respectively). It cannot be argued that the individual learning scores approaching 1.0 for the model are overestimates. For example, although the bobbing response to CS° is low compared to *P*_resp_, the bobbing response to CS^+^ is also comparatively low (Figures 4A,B). Furthermore, the majority of individuals showing a maximum *P*_resp_ of >0.95 to CS° also show bobbing (8 of 10 animals), whereas most individuals with a low *P*_resp_ towards CS^+^ (<0.5) do not show bobbing (12 of 14 animals). This correlation reflects the fact that bobbing is a key predictive variable in the model. Despite this, the large difference between the *model-LI* and the *bobbing-LI* is mainly due to a comparatively large number of animals with zero or small bobbing scores (−0.05 to 0.05), resulting in a skewed distribution with a mode at zero, whereby outliers with exceptionally high scores tamp down all lower scores (Figure 4D). It should also be noted that bobbing appears to be a weaker indicator of learning than other, more commonly used single metrics in crickets. For example, in a similar though not entirely comparable, differential, olfactory appetitive conditioning paradigm, in which the times spent visiting a rewarded and non-rewarded odour source are evaluated, crickets show a mean performance index of around 30, 2 h after 2 training trials (Figure 3B, white bar in Matsumoto and Mizunami, 2002). This corresponds to a mean *LI* of 0.3 on our scale, compared to a *bobbing-LI* of 0.14 and a *model-LI* of 0.42, when the means are taken, rather than the medians. Nonetheless, one of the main advantages of the model is that it captures animals that show no bobbing as having learnt. This reflects the impact of the additional variables in the model, and confirms the complexity of a conditioned response (cf. Onodera et al., 2019). The fact that we could also construct a model excluding bobbing (*Model-4*) with an average predictive power of 87% also confirmed that general movement variables are valid proxies for measuring searching behaviour that typify a conditioned response. Our findings thus exemplify the key benefit of using binary regression analysis, which also encompasses interactions among the behavioural variables in relation to *P*_resp_, the compound measure (Cullen et al., 2012). Nonetheless, a potential drawback is that the influence of any environmental or genetic factor with antagonistic effects on different model components may be underestimated. To rule this out in future studies, it will be important to routinely check for effects on each individual parameter, in addition to the model metric.

A notable difference between the two learning indexes is the distribution of the individual data points (Figures 4C,D). Contrasting the skewed distribution for the *bobbing-LI*, the model data has two modes at 0 and 1.0, with a just significant coefficient of bimodality (Pfister et al., 2013). It seems unlikely that this distribution is simply a result of binary regression analysis, with its maximal possible extreme values of 0 and 1. Firstly, as is characteristic for binary regression, a combined plot of the individual *P*_resp_ values for the CS° and CS^+^ arranged in ascending order yields a sigmoid curve with many 0 and 1 scores, but also many, evenly distributed intermediate scores (Supplementary Figure 1A). Secondly, a corresponding plot for the *model-LI* (the difference between the *P*_resp_ values for the CS° and CS^+^) in contrast, yields a more linear distribution, with relatively evenly spaced positive and negative values (Supplementary Figure 1B). This illustrates that our model has the potential to effectively capture inter-individual differences in learning. However, while our data suggest a broad spectrum of learning capacity, with seemingly very poor and excellent learners, the key question, which now needs to be addressed, is whether such differences are consistent over time, and thus indicative of a biologically meaningful “personality” trait for learning in crickets? This seems likely, considering that a similar situation has been reported for classical conditioning of honey bees, where the population comprises two types of animals, those that have acquired a conditioned response, and those that have not (Pamir et al., 2011). Furthermore, we have already demonstrated that crickets taken from standard breeding colonies show consistent inter-individual differences with respect to their general motility, exploratory behaviour, aggressiveness and their decision to approach or avoid a novel stimulus, resulting in basically two ethotypes comprising aggressive-proactive and submissive-reactive individuals (Rose et al., 2017; Balsam and Stevenson, 2020, 2021). Notably, these differences appear to arise as a result of social experience, in particular social subjugation by aggressive adult males during development (Balsam and Stevenson, 2020, 2021). Accordingly, we are currently testing the hypothesis, that aggressive social experience has a major impact on individual learning capacity.

## Data Availability Statement

The raw data supporting the conclusions of this article will be made available by the authors, without undue reservation.

## Author Contributions

PS and KB contributed to conception and methodology of the experiments, analysed the data, and wrote the original draft of the article. PS acquired the funding and supervised the project. KB performed the experiments and collected the data. All authors contributed to discussing the results, writing the manuscript, and approved the submitted version.

## Funding

The authors acknowledge support from the German Research Foundation (DFG, STE 714/5-1) and Leipzig University within the program of Open Access Publishing.

## Conflict of Interest

The authors declare that the research was conducted in the absence of any commercial or financial relationships that could be construed as a potential conflict of interest.

## Publisher's Note

All claims expressed in this article are solely those of the authors and do not necessarily represent those of their affiliated organizations, or those of the publisher, the editors and the reviewers. Any product that may be evaluated in this article, or claim that may be made by its manufacturer, is not guaranteed or endorsed by the publisher.

## Supplementary Material

Supplementary Figure 1 Plots of individual P_resp_ values **(A)** for the non-rewarded and the rewarded odour (CS° and blue circles, respectively CS^+^ and purple circles) and the resultant learning indexes **(B)** derived from the binary regression *Model-1* and sorted in ascending magnitude.
