# Supplementary figures and images for: Individual Scores for Associative Learning in a Differential Appetitive Olfactory Paradigm Using Binary Logistic Regression Analysis
Source: Front Behav Neurosci. 2021 Sep 28;15:741439. doi: 10.3389/fnbeh.2021.741439 (PMC8505765; doi:10.3389/fnbeh.2021.741439)

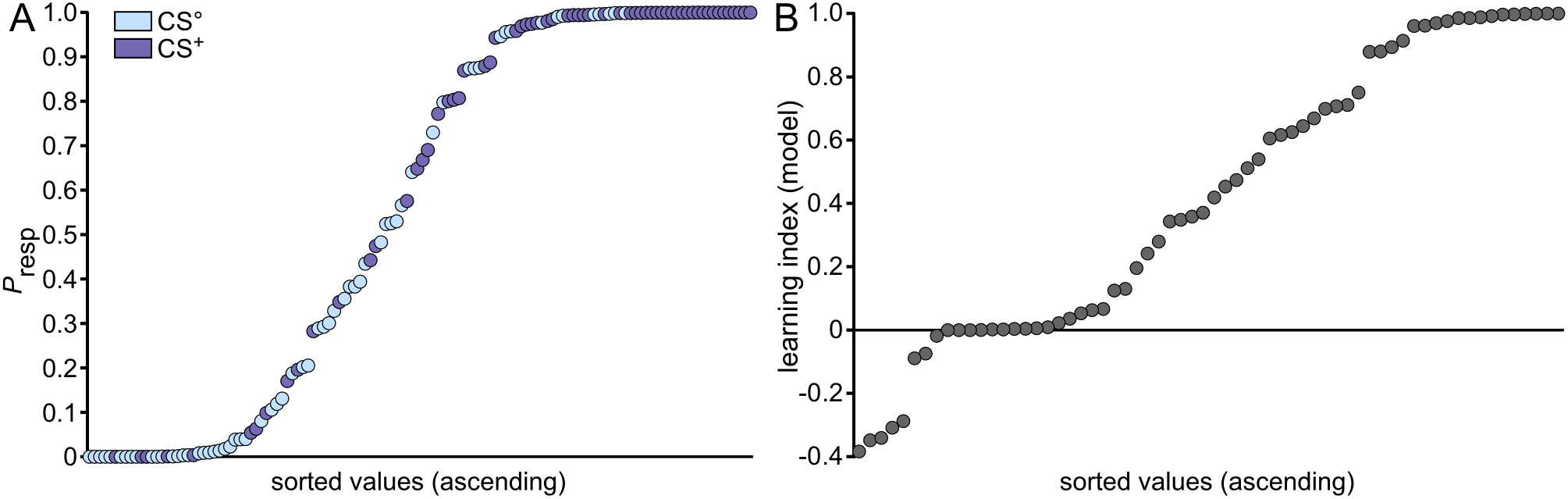

Supplement: Supplementary Figure 1 — Plots of individual Presp values (A) for the non-rewarded and the rewarded odour (CS° and blue circles, respectively CS+ and purple circles) and the resultant learning indexes (B) derived from the binary regression Model-1 and sorted in ascending magnitude. [file Image_1.TIF]
